# Preclinical and Molecular Docking Insights into the Chemopreventive Role of Fenugreek Seed Extract in a Murine Model of Colorectal Cancer

**DOI:** 10.3390/ph18040490

**Published:** 2025-03-28

**Authors:** Arif Khan, Khaled S. Allemailem, Arwa Essa Alradhi, Faizul Azam

**Affiliations:** 1 Department of Basic Health Sciences, College of Applied Medical Sciences, Qassim University, Buraydah 51452, Saudi Arabia; 2 Department of Medical Laboratories, College of Applied Medical Sciences, Qassim University, Buraydah 51452, Saudi Arabia; k.allemailem@qu.edu.sa; 3 General Administration for Infectious Disease Control, Ministry of Health, Riyadh 12382, Saudi Arabia; aealradhi@moh.gov.sa; 4 Department of Medicinal Chemistry and Pharmacognosy, College of Pharmacy, Qassim University, Buraydah 51452, Saudi Arabia; f.azam@qu.edu.sa

**Keywords:** colorectal cancer, animal model, in vivo study, drug design, cancer therapy, molecular docking simulation

## Abstract

**Background/Objectives:** Colorectal cancer (CRC) remains a leading cause of cancer-related mortality, necessitating the development of effective preventive strategies. Fenugreek (*Trigonella foenum-graecum*) possesses well-documented pharmacological properties; however, its chemopreventive potential in colorectal cancer (CRC) remains unexplored. This study evaluates the efficacy of methanolic fenugreek seed extract (FSE) in an azoxymethane (AOM)-induced murine colorectal cancer (CRC) model, focusing on the modulation of oxidative stress, regulation of biomarkers, induction of apoptosis, and maintenance of epithelial integrity. **Methods:** FSE was extracted using cold maceration (yield: 24%) and analyzed by gas chromatography–mass spectrometry (GC-MS), identifying 13 bioactive compounds, including benzene, 1,3-dimethyl-; 1,3-cyclopentadiene, 5-(1-methylethylidene)-; o-Xylene; benzenepropanoic acid, 3,5-bis(1,1-dimethylethyl)-4-hydroxy-; and benzene, 1,2,3-trimethyl-. All 13 compounds identified were matched with the NIST library with high confidence. Molecular docking was used to assess the interactions of FSE bioactives with E-cadherin–β-catenin complexes. Swiss albino mice received an FSE pre-treatment before AOM induction and continued this treatment three times weekly for 21 weeks. Key assessments included survival analysis, body weight changes, serum biomarker levels (GGT, 5′-NT, LDH), antioxidant enzyme activities (SOD, CAT, GPx1, MDA), reactive oxygen species (ROS) quantification, apoptosis detection via flow cytometry, and immunofluorescence-based evaluation of E-cadherin dynamics. **Results:** FSE improved survival rates, mitigated AOM-induced weight loss, and dose-dependently reduced serum biomarker levels. Antioxidant enzyme activity was restored, while MDA levels declined. A dose-dependent increase in ROS facilitated apoptosis, as confirmed by flow cytometry (16.7% in the low-dose FSE group and 34.5% in the high-dose FSE group). Immunofluorescence studies revealed that FSE-mediated restoration of E-cadherin localization counteracted AOM-induced epithelial disruptions. **Conclusions:** FSE exhibits potent chemopreventive potential against CRC by modulating oxidative stress, regulating key biomarkers, inducing apoptosis, and restoring epithelial integrity. These findings support further investigations into its clinical relevance for CRC prevention.

## 1. Introduction

Cancer remains one of the most significant global health challenges, with incidence and mortality rates steadily increasing worldwide. According to the GLOBOCAN 2022 report, it was estimated that 20 million new cancer cases and 10 million cancer-related deaths occurred globally in 2022, marking a substantial rise from previous years. These statistics highlight the urgent need for innovative cancer prevention, diagnosis, and treatment strategies. Among various malignancies, colorectal cancer (CRC) continues to be a primary contributor to the global cancer burden, ranking as the third most frequently diagnosed cancer and the second leading cause of cancer-related mortality. In 2022 alone, CRC accounted for approximately 1.93 million new cases and 903,859 deaths worldwide, emphasizing the necessity of developing effective preventive and therapeutic interventions [1,2].

The burden of CRC is not uniform across regions, revealing significant disparities in incidence and mortality rates. Historically, high-income countries, such as those in North America, Europe, and Australia, have reported the highest CRC rates, largely due to dietary habits characterized by a high intake of red and processed meats, low fiber consumption, obesity, and a sedentary lifestyle. However, in recent years, a rapid rise in CRC incidence has been observed in low- and middle-income countries (LMICs), particularly in Asia, where urbanization and shifts toward Western dietary patterns have contributed to an increase in cases. Countries like China and India now report CRC incidence rates comparable to those in high-income nations [3,4,5]. In Saudi Arabia, CRC has become one of the most frequently diagnosed cancers, with the Saudi Cancer Registry documenting a consistent rise in cases over the past decade. This increasing prevalence is attributed to lifestyle changes, high-calorie and low-fiber diets, physical inactivity, and genetic predispositions, underscoring an urgent need for prevention and early intervention strategies [6,7,8].

The etiology of CRC is complex and influenced by genetic, environmental, and lifestyle factors. Although hereditary syndromes such as familial adenomatous polyposis (FAP) and Lynch syndrome account for a small proportion of cases, the vast majority of CRC instances are sporadic and driven by modifiable risk factors. Diet plays a crucial role; a high consumption of processed meats and low intake of fruits and vegetables have been associated with an increased risk of CRC [9,10,11]. Additionally, obesity, physical inactivity, smoking, and alcohol consumption have been identified as significant lifestyle contributors to the development of CRC [12]. Chronic inflammation, particularly in inflammatory bowel disease (IBD), further exacerbates the risk by promoting DNA damage, cellular proliferation, and tumorigenesis [13]. Recent research has also implicated dysbiosis of the gut microbiome in the pathogenesis of CRC, with microbial imbalances linked to heightened inflammatory responses and carcinogenesis [14].

Despite advancements in CRC screening, early detection, and treatment strategies, the disease poses a significant challenge due to high recurrence rates and limited therapeutic options in its advanced stages [15,16,17]. Surgical resection, chemotherapy, and radiotherapy remain the cornerstones of CRC management. However, chemotherapy regimens such as FOLFOX (5-fluorouracil, leucovorin, oxaliplatin) and FOLFIRI (5-fluorouracil, leucovorin, irinotecan) often lead to severe adverse effects, including neuropathy, myelosuppression, and gastrointestinal toxicity, which significantly impact patient quality of life and adherence to treatment [18]. Furthermore, the emergence of drug resistance mechanisms limits the long-term effectiveness of chemotherapy, emphasizing the need for safer, more effective therapeutic alternatives [19,20]. This has sparked interest in plant-derived bioactive compounds as potential chemopreventive and therapeutic agents for CRC [21,22].

Natural products have demonstrated significant promise in cancer prevention and treatment due to their ability to modulate key signaling pathways involved in tumor progression while exhibiting lower toxicity profiles than conventional therapies [23]. Several plant-derived compounds have demonstrated potent anticancer properties against colorectal cancer (CRC). Curcumin, a bioactive polyphenol derived from turmeric, has been extensively studied for its ability to inhibit NF-κB and STAT3 signaling, thereby suppressing the proliferation and metastasis of CRC [24,25]. Similarly, epigallocatechin gallate (EGCG) from green tea has been reported to induce apoptosis and inhibit CRC cell growth by activating caspase-3 and PARP while downregulating STAT3 expression, disrupting pro-survival signaling in cancer cells [26]. Resveratrol, a polyphenolic compound found in grapes, has demonstrated strong anticancer potential by modulating multiple molecular pathways involved in cancer development. It exerts its effects by reducing oxidative stress, inducing apoptosis through the activation of both intrinsic and extrinsic apoptotic pathways, and inhibiting angiogenesis, thereby restricting tumor growth and metastasis [27]. Our research group has made significant contributions to exploring novel plant-based interventions for CRC prevention and therapy. We previously demonstrated the chemosensitizing effect of liposomal diallyl disulfide (DADS) in combination with low-dose liposomal oxaliplatin, resulting in enhanced apoptosis through ROS induction in CRC cells. Furthermore, we reported the chemopreventive and chemosensitizing potential of liposomal diallyl trisulfide (DATS) in an AOM-induced CRC animal model. Pre-treatment with liposomal DATS, in combination with liposomal doxorubicin, not only prevented liver and lung metastasis but also effectively inhibited CRC progression, thereby suppressing carcinogenesis in the colon. Additionally, our research group has also reported the chemopreventive effects of methanolic extract of *Artemisia annua* in CRC models, demonstrating its efficacy in both in vitro and in vivo systems. These findings reinforce the therapeutic potential of natural compounds as adjuncts to conventional CRC therapies, warranting further clinical investigation into their translational application [28,29,30].

Among medicinal plants with notable anticancer properties, fenugreek (*Trigonella foenum-graecum*) has gained increasing attention for its potential role in cancer prevention and therapy [31]. A leguminous plant native to the Mediterranean region and South Asia, fenugreek has been traditionally used for its anti-inflammatory, antioxidant, and metabolic-regulating properties. Recent studies have highlighted its anticancer potential, primarily attributed to its rich phytochemical composition, including diosgenin, 4-hydroxyisoleucine, flavonoids, and saponins. These bioactive compounds exert multiple anticancer effects, including inducing apoptosis, arresting the cell cycle, and modulating key oncogenic pathways, such as the NF-κB and PI3K/Akt signaling pathways. Various cancer models have demonstrated fenugreek’s antitumor potential, including its ability to inhibit proliferation, suppress metastasis, and enhance chemosensitivity [32,33,34,35]. Our research has further demonstrated that methanolic fenugreek seed extract (FSE) induces p53-dependent mitotic catastrophe in breast cancer cells, resulting in apoptosis and reinforcing its role as a potent anticancer agent. Additionally, fenugreek seed extracts possess strong antioxidant properties, enhancing the activity of superoxide dismutase (SOD), catalase (CAT), and glutathione peroxidase (GPx1) while significantly reducing malondialdehyde (MDA) levels, a key marker of oxidative stress [36]. By mitigating oxidative damage, fenugreek may contribute to cancer prevention and therapy, further supporting its potential as a natural chemopreventive agent. However, despite promising findings in breast, liver, and other cancer models, its precise molecular mechanisms across different malignancies remain to be fully elucidated, warranting further investigation.

Despite promising in vitro evidence supporting fenugreek’s anticancer effects, limited in vivo studies have been conducted to evaluate its efficacy in CRC models. Furthermore, the precise molecular mechanisms underlying its chemopreventive properties remain largely unexplored. Molecular docking, an advanced computational technique widely used in drug discovery, provides valuable insights into the interactions between bioactive compounds and their target proteins, allowing for the identification of potential therapeutic mechanisms. Previous studies have successfully employed molecular docking to predict interactions between natural compounds and oncogenic targets, such as thymoquinone in lung cancer and diallyl sulfide derivatives in CRC [29,30,37,38]. Given the diverse array of bioactive compounds in fenugreek, molecular docking provides an opportunity to elucidate its specific molecular targets in colorectal cancer (CRC) pathogenesis.

The increasing global burden of CRC and the limitations of conventional therapies necessitate the exploration of novel, safer, and more effective interventions. Fenugreek (*Trigonella foenum-graecum*), a medicinal plant with well-documented pharmacological properties and a favorable safety profile, has shown promising anticancer potential. However, its precise molecular mechanisms in CRC prevention remain largely unexplored. The present study aims to bridge this gap by investigating the chemopreventive potential of methanolic FSE and elucidating its role in the suppression of CRC. Given its diverse bioactive constituents, we hypothesize that FSE exerts significant chemopreventive effects against azoxymethane (AOM)-induced colorectal carcinogenesis by modulating oxidative stress, restoring epithelial integrity, regulating tumor-associated biomarkers, and inducing apoptosis in transformed cells. We employ an in vivo AOM-induced CRC model, along with molecular docking analyses, to test this hypothesis and uncover the mechanistic basis of FSE’s anticancer effects. By integrating computational and preclinical approaches, this research aims to provide mechanistic insights into how FSE modulates key oncogenic pathways, influences tumor progression, and regulates oxidative stress and apoptosis in colorectal cancer (CRC). Understanding these molecular interactions may offer new avenues for plant-based strategies in CRC management. The findings of this study can serve as a foundation for future translational research, contributing to the development of fenugreek-derived therapeutic agents as potential adjuncts or alternatives to conventional treatments for colorectal cancer (CRC).

## 2. Results

### 2.1. Yield Percentage of FSE and GC-MS Analysis

**Figure 1:**
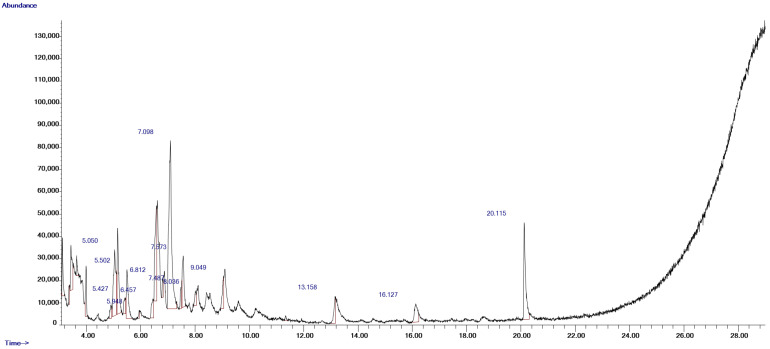
GC-MS chromatogram illustrating bioactive compounds identified in methanolic fenugreek seed extract (FSE) prepared using cold maceration method.

Methanolic extraction of fenugreek seeds (FSE) yielded 42%, producing 72 g of dried extract. GC-MS analysis identified 13 bioactive compounds, including benzene, 1,3-dimethyl-; 1,3-cyclopentadiene, 5-(1-methylethylidene)-; o-Xylene; and benzene, 1,2,3-trimethyl-, benzenepropanoic acid, 3,5-bis(1,1-dimethylethyl)-4-hydroxy- (Figure 1, Table 1). All 13 compounds identified were matched with the NIST library with high confidence, as detailed in Table 1, with most compounds showing high-confidence matches (over 90%). These compounds exhibit antioxidant, anti-inflammatory, antimicrobial, and cytotoxic properties, consistent with the chemopreventive effects observed in FSE-treated groups [39,40,41,42].

### 2.2. Effect of FSE on AOM-Induced Average Body Weight and Survival Rates

**Figure 2:**
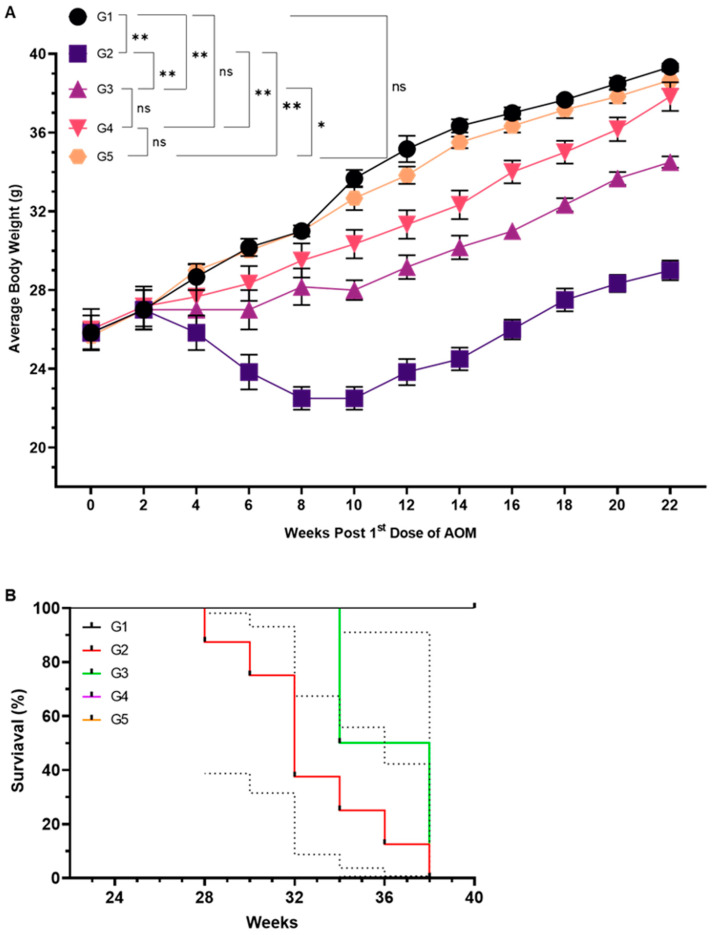
Effect of FSE on AOM-induced changes in average body weight and survival rates. (**A**) Average body weight (ABW) changes across experimental groups during study period. Data are presented as mean with 95% confidence intervals (CI), based on sample size of five mice per group (*n* = 5). (**B**) Kaplan–Meier survival curves illustrating survival rates monitored up to week 40, with sample size of ten mice per group (*n* = 10, all survival rates of G1, G4, G5 are in the range of 100%). Statistical significance was determined using one-way ANOVA followed by Tukey’s post hoc testing for ABW and log-rank test for survival analysis. ‘ns’ indicates no significant difference. Asterisks denote levels of statistical significance: * *p* < 0.05 and ** *p* < 0.01.

A significant decline in average body weight (ABW) was observed in Group 2 (G2), which received AOM without FSE treatment. The ABW in G2 decreased from 30.2 g at baseline to 22.5 g by week 8, followed by partial recovery to 29.0 g by week 22. However, at week 22, the ABW in G2 remained significantly lower than that of G1 (*p* < 0.1), G3 (*p* < 0.01), G4 (*p* < 0.01), and G5 (*p* < 0.01), all of which maintained higher and more stable ABWs, with G1 and G5 around 38.5 g and 38.66 g, respectively (Figure 2A). FSE treatment mitigated AOM-induced weight loss in a dose-dependent manner. G4, which received a high dose of FSE, exhibited a stable ABW of 37.83 g, comparable to that of G1 and G5, suggesting a protective effect against AOM-induced weight reduction. In contrast, G3, which received a lower dose of FSE, experienced moderate weight loss but remained significantly higher than G2 (*p* < 0.01). Survival analysis further highlighted the protective effects of FSE. The Kaplan–Meier survival curve (Figure 2B) indicated that G2 exhibited the highest mortality rate (80%), whereas G3 demonstrated a substantially lower mortality rate (20%). Notably, G4 exhibited complete protection, with a 100% survival rate, underscoring the dose-dependent protective role of FSE in mitigating AOM-induced mortality.

### 2.3. Effect of FSE on Serum Cancer Marker Enzymes

**Figure 3:**
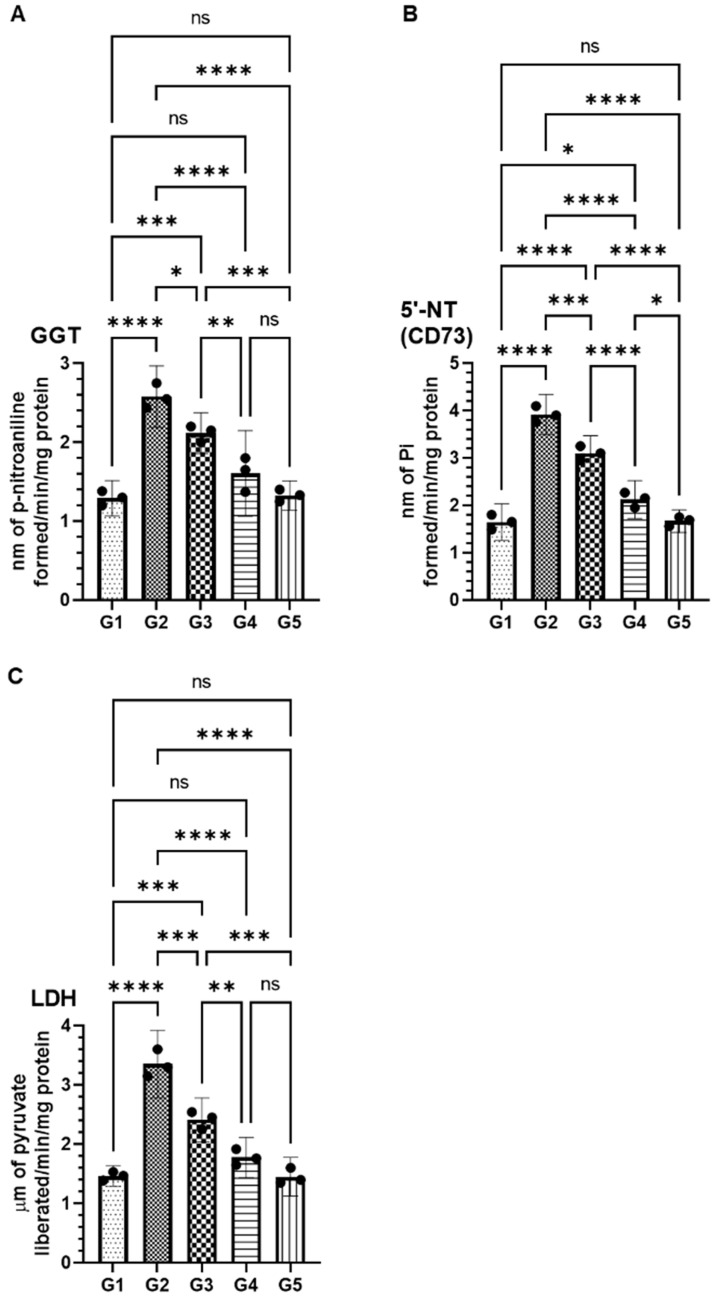
Modulatory effects of FSE on serum cancer marker enzymes altered by AOM exposure. This figure illustrates effect of fenugreek seed extract (FSE) on serum levels of key cancer biomarkers in AOM-induced colorectal cancer model. Panels represent activity levels of (**A**) gamma-glutamyl transferase (GGT), (**B**) 5′-nucleotidase (CD73), and (**C**) lactate dehydrogenase (LDH) across experimental groups. AOM exposure (G2) significantly elevated levels of these enzymes compared to control group (G1), indicating metabolic dysregulation associated with tumor progression. FSE treatment, particularly at higher doses (G4), effectively reduced these enzyme levels toward baseline values in dose-dependent manner. FSE-only group (G5) showed no significant deviation from control, confirming safety of extract under normal physiological conditions. Data are presented as means with error bars representing 95% confidence interval (CI) from three independent experiments. Symbols indicate statistical significance: * *p* < 0.05, ** *p* < 0.01, *** *p* < 0.001, and **** *p* < 0.0001; ’ns’ denotes no significant difference between groups.

Serum enzyme analysis revealed significant alterations in key cancer biomarkers following AOM exposure, which were effectively modulated by FSE treatment. In the AOM-only group (G2), levels of gamma-glutamyl transferase (GGT), 5′-nucleotidase (CD73), and lactate dehydrogenase (LDH) were markedly elevated to 2.58 nmol (95% CI: 2.19–2.97), 3.92 nmol (95% CI: 3.49–4.34), and 3.35 µmol (95% CI: 2.78–3.91), respectively, compared to 1.29 nmol (95% CI: 1.06–1.51), 1.64 nmol (95% CI: 1.26–2.03), and 1.43 µmol (95% CI: 1.28–1.63) in the vehicle control group (G1) (Figure 3). FSE administration significantly attenuated these pathological alterations in a dose-dependent manner. In the low-dose FSE group (G3), GGT, CD73, and LDH levels were reduced to 2.11 nmol (95% CI: 1.85–2.37), 3.10 nmol (95% CI: 2.72–3.47), and 2.41 µmol (95% CI: 2.05–2.78), respectively (Figure 3). A more pronounced reduction was observed in the high-dose FSE group (G4), where GGT, CD73, and LDH levels approached near-baseline values at 1.60 nmol (95% CI: 1.06–2.14), 2.12 nmol (95% CI: 1.72–2.52), and 1.77 µmol (95% CI: 1.43–2.11), respectively (Figure 3). Notably, the FSE-only group (G5) exhibited biomarkers comparable to G1, indicating that FSE administration alone did not interfere with normal physiological processes. These findings underscore the chemopreventive potential of FSE in mitigating AOM-induced biochemical dysregulation and restoring homeostasis.

### 2.4. Modulation of Antioxidant Enzymes by FSE in AOM-Exposed Colonic Tissues

**Figure 4:**
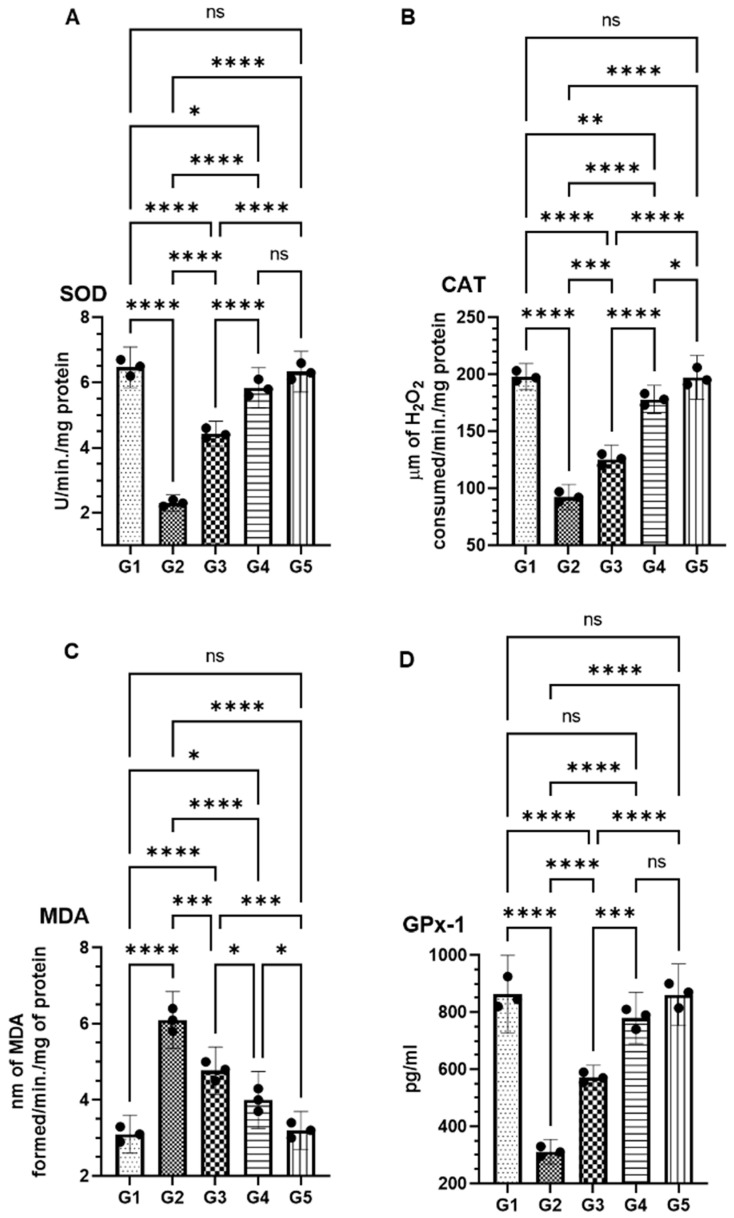
Effects of FSE on antioxidant enzyme activities in AOM-exposed colonic tissues. This figure illustrates impact of fenugreek seed extract (FSE) on antioxidant defense mechanisms in colon tissues of AOM-induced colorectal cancer (CRC) mice. Panels depict activity levels of (**A**) superoxide dismutase (SOD); (**B**) catalase (CAT); (**C**) malondialdehyde (MDA), a marker of lipid peroxidation; and (**D**) glutathione peroxidase 1 (GPx1) across experimental groups. AOM exposure (G2) significantly impaired antioxidant enzyme activity, as evidenced by reduced SOD, CAT, and GPx1 levels, alongside elevated MDA concentrations, indicating increased oxidative stress and lipid peroxidation. FSE treatment resulted in dose-dependent restoration of antioxidant enzyme activity, with high-dose group (G4) achieving levels comparable to those of control group (G1), indicating potent protective effect against AOM-induced oxidative stress. FSE-only group (G5) showed no significant deviations from G1, confirming safety of extract under normal physiological conditions. Data are presented as means with error bars representing 95% confidence interval (CI) from three independent experiments. Statistical significance is indicated by * *p* < 0.05, ** *p* < 0.01, *** *p* < 0.001, and **** *p* < 0.0001; ‘ns’ denotes no significant difference between groups.

FSE treatment effectively restored the significantly impaired antioxidant enzyme activity caused by AOM exposure. Superoxide dismutase (SOD), a key regulator of oxidative stress, was markedly reduced in the AOM-only group (G2) to 2.30 U (95% CI: 2.05–2.54) compared to 6.46 U (95% CI: 5.84–7.09) in the control group (G1). FSE treatment led to a dose-dependent restoration, with SOD activity reaching 4.43 U (95% CI: 4.05–4.81) in the low-dose group (G3) and 5.83 U (95% CI: 5.20–6.45) in the high-dose group (G4), approaching levels observed in G1 (Figure 4A). Catalase (CAT) activity showed a similar trend. AOM exposure significantly reduced CAT levels to 92.33 µmol (95% CI: 81.13-103.53), while FSE treatment increased activity to 125 µmol (95% CI: 112.83–137.90) in G3 and 178.00 µmol (95% CI: 165.58–190.42) in G4, indicating effective oxidative stress mitigation (Figure 4B). Malondialdehyde (MDA), a marker of lipid peroxidation, was also significantly reduced with FSE treatment. Elevated MDA levels in G2 (6.10 nmol; 95% CI: 5.35-6.84) decreased to 4.76 nmol (95% CI: 4.14–5.39) in G3 and 4.00 nmol (95% CI: 3.25–4.74) in G4, suggesting FSE’s role in preserving cellular integrity by limiting oxidative damage (Figure 4C). Glutathione peroxidase (GPx1), essential for detoxifying hydrogen peroxide, was significantly depleted in G2 (311.66 pg; 95% CI: 268.04–355.28). FSE treatment restored GPx1 levels to 571.66 pg (95% CI: 528.04–615.28) in G3 and 780.00 pg (95% CI: 690.43–869.56) in G4, aligning closely with control group levels (863.33 pg; 95% CI: 727.08–999.58) (Figure 4D).

Notably, the FSE-only group (G5) showed no significant differences from G1 across all antioxidant parameters, indicating that FSE did not disrupt normal physiological processes. These results underscore the dose-dependent capacity of FSE to restore antioxidant defenses and mitigate AOM-induced oxidative stress.

### 2.5. Assessment of Reactive Oxygen Species in Colonic Cells by DCFDA Flow Cytometry

**Figure 5:**
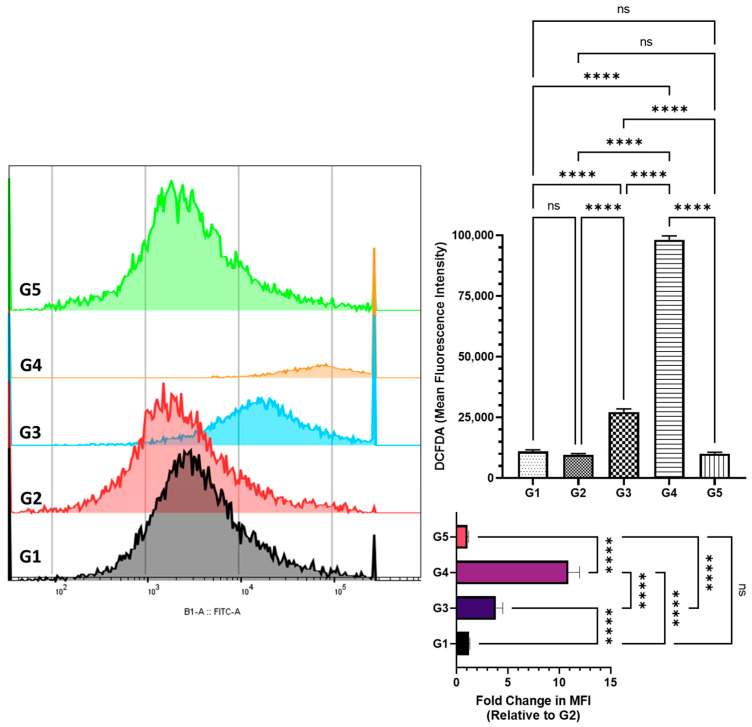
An evaluation of FSE’s impact on reactive oxygen species (ROS) levels in colonic cells. This figure illustrates the effects of fenugreek seed extract (FSE) on intracellular reactive oxygen species (ROS) levels in an AOM-induced colorectal cancer (CRC) model, as assessed using the 2′,7′-dichlorofluorescein diacetate (DCFDA) assay via flow cytometry. The mean fluorescence intensity (MFI) values reflect ROS generation across the experimental groups. AOM exposure (G2) did not induce a significant increase in ROS compared to the control group (G1), suggesting a potential adaptive cellular response during tumor progression. In contrast, FSE treatment resulted in a significant, dose-dependent increase in ROS levels, with the low-dose group (G3) and high-dose group (G4) exhibiting marked increases compared to the control group (G2). This ROS elevation indicates FSE’s role in promoting oxidative-stress-mediated cytotoxicity in transformed cells. The FSE-only group (G5) exhibited ROS levels comparable to the control group, confirming that FSE alone does not induce oxidative stress under normal physiological conditions. The data are presented as means with error bars representing the 95% confidence interval (CI) from three independent experiments. Statistical significance is indicated by (****) *p* < 0.0001; ’ns’ denotes no significant difference between groups.

**Figure 6:**
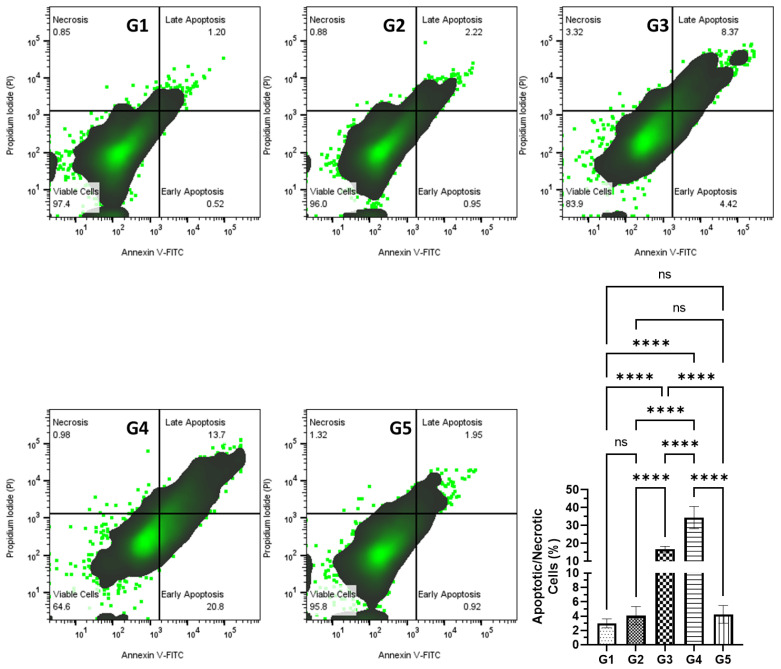
An analysis of FSE-induced apoptosis in colonic cells assessed by Annexin V-FITC/PI flow cytometry. This figure illustrates the pro-apoptotic effects of fenugreek seed extract (FSE) in colonic cells from an AOM-induced colorectal cancer (CRC) model, as evaluated using Annexin V-FITC and propidium iodide (PI) staining, analyzed using flow cytometry. The data distinguish between viable, early apoptotic, late apoptotic, and necrotic cells across the experimental groups. AOM exposure (G2) induced minimal apoptosis, indicating that carcinogen-exposed cells failed to initiate intrinsic cell death pathways. In contrast, FSE treatment significantly increased apoptosis in a dose-dependent manner, with the low-dose group (G3) showing a notable rise in apoptotic cells and the high-dose group (G4) exhibiting a marked apoptotic fraction of 34.5%, indicating robust activation of programmed cell death mechanisms. The FSE-only group (G5) demonstrated apoptosis rates comparable to the control group (G1), confirming FSE’s safety under non-pathological conditions. The data are presented as means with error bars representing the 95% confidence interval (CI) from three independent experiments. Statistical significance is indicated by (****) *p* < 0.0001; ’ns’ denotes no significant difference between groups.

Reactive oxygen species (ROS) levels in colonic cells were measured using DCFDA staining to evaluate oxidative stress. The AOM-treated group (G2) showed minimal oxidative stress induction, with a mean fluorescence intensity (MFI) of 9000 (95% CI: 8296–9705), suggesting an adaptive cellular response during tumor progression (Figure 5). ROS levels in G2 were statistically similar to the control (G1) (MFI: 10,993; 95% CI: 10,123–11,863) and the FSE-only group (G5) (MFI: 9610; 95% CI: 8421–10,799), indicating that FSE alone did not induce oxidative stress. In contrast, FSE pre-treatment resulted in a significant, dose-dependent increase in ROS levels, suggesting its potential role in inducing oxidative-stress-mediated cytotoxicity in transformed cells. The low-dose FSE group (G3) exhibited elevated ROS levels (MFI: 34,850; 95% CI: 30,925–38,775), while the high-dose group (G4) showed a more pronounced increase (MFI: 98,233; 95% CI: 81,650–114,815). Compared to G2, ROS levels in G3 and G4 increased 3.84-fold (95% CI: 3.18–4.50) and 10.90-fold (95% CI: 9.84–11.97), respectively. These results suggest that FSE induces controlled oxidative stress, potentially facilitating apoptosis in transformed cells. While FSE enhanced antioxidant defenses in earlier assessments, it simultaneously elevated ROS levels in G3 and G4, reflecting the biphasic role of ROS in cancer biology. ROS support cell survival at low levels, but excessive accumulation triggers apoptosis. Flow cytometry confirmed a significant increase in apoptosis in FSE-treated groups (G3 and G4) (Figure 6). This dual regulatory effect underscores FSE’s potential as a chemopreventive agent, effectively balancing oxidative homeostasis to suppress tumor progression in colonic carcinogenesis.

### 2.6. Assessment of Apoptosis in Colonic Cells via Annexin V-FITC-PI Flow Cytometry

The pro-apoptotic potential of FSE was assessed through Annexin V-FITC and propidium iodide (PI) staining, which enabled the distinction between viable, early apoptotic, late apoptotic, and necrotic cells. Flow cytometric analysis revealed a substantial increase in apoptotic cell populations in the FSE-treated groups, further substantiating its apoptosis-inducing effects (Figure 6). In the high-dose FSE group (G4), the apoptotic fraction surged to 34.5% (95% CI: 28.30–40.71), reflecting a strong activation of programmed cell death pathways in response to FSE treatment. Similarly, the low-dose FSE group (G3) exhibited a significant apoptotic rate of 16.7% (95% CI: 15.10–18.40), indicating a dose-dependent pro-apoptotic effect. Conversely, the AOM-only group (G2) displayed negligible apoptosis, highlighting the failure of damaged cells to initiate intrinsic cell death mechanisms following carcinogen exposure. The marked apoptotic response in the FSE-treated groups suggests that FSE-mediated ROS accumulation is a crucial upstream event in the induction of apoptosis, likely engaging both intrinsic (mitochondrial) and extrinsic (death receptor) pathways. This selective induction of apoptosis in pre-neoplastic and neoplastic cells underscores the chemopreventive efficacy of FSE in counteracting AOM-induced tumorigenesis. Collectively, these findings highlight the mechanistic role of FSE as a dynamic modulator of oxidative-stress-driven apoptosis, facilitating the selective elimination of transformed cells while preserving the integrity of normal tissues.

### 2.7. Immunofluorescence Analysis of E-Cadherin and Phosphorylated E-Cadherin Localization in Colonic Epithelial Tissues

**Figure 7:**
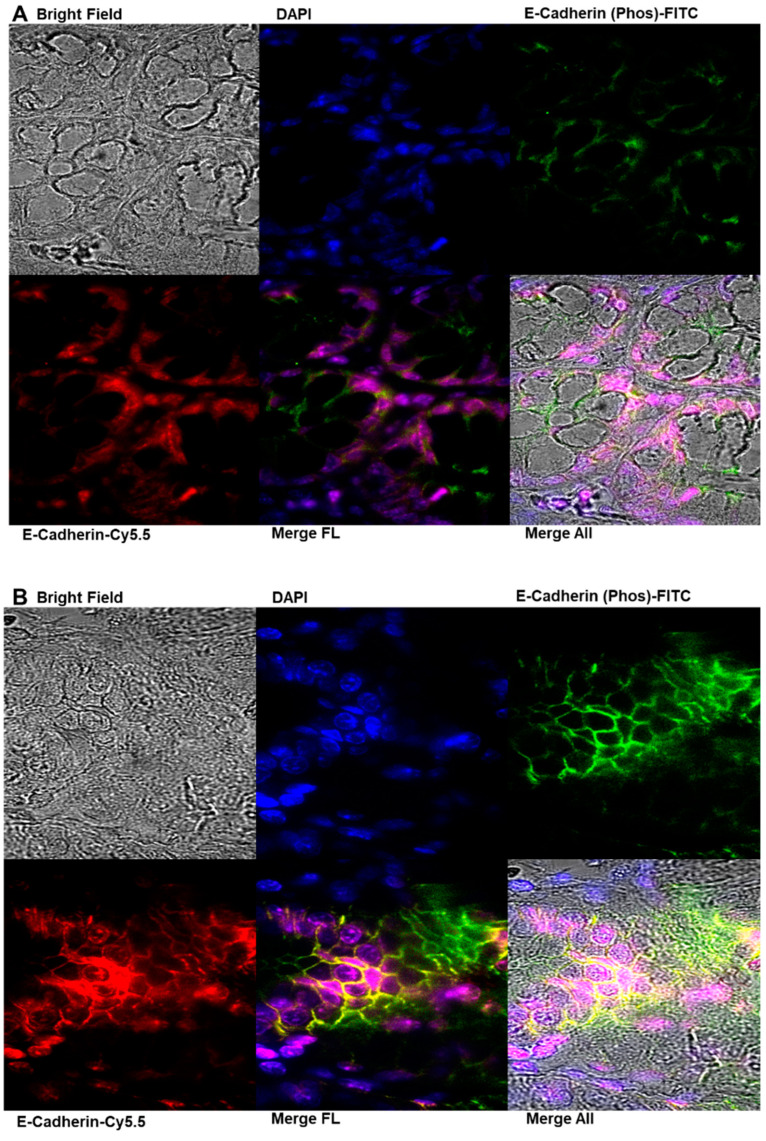

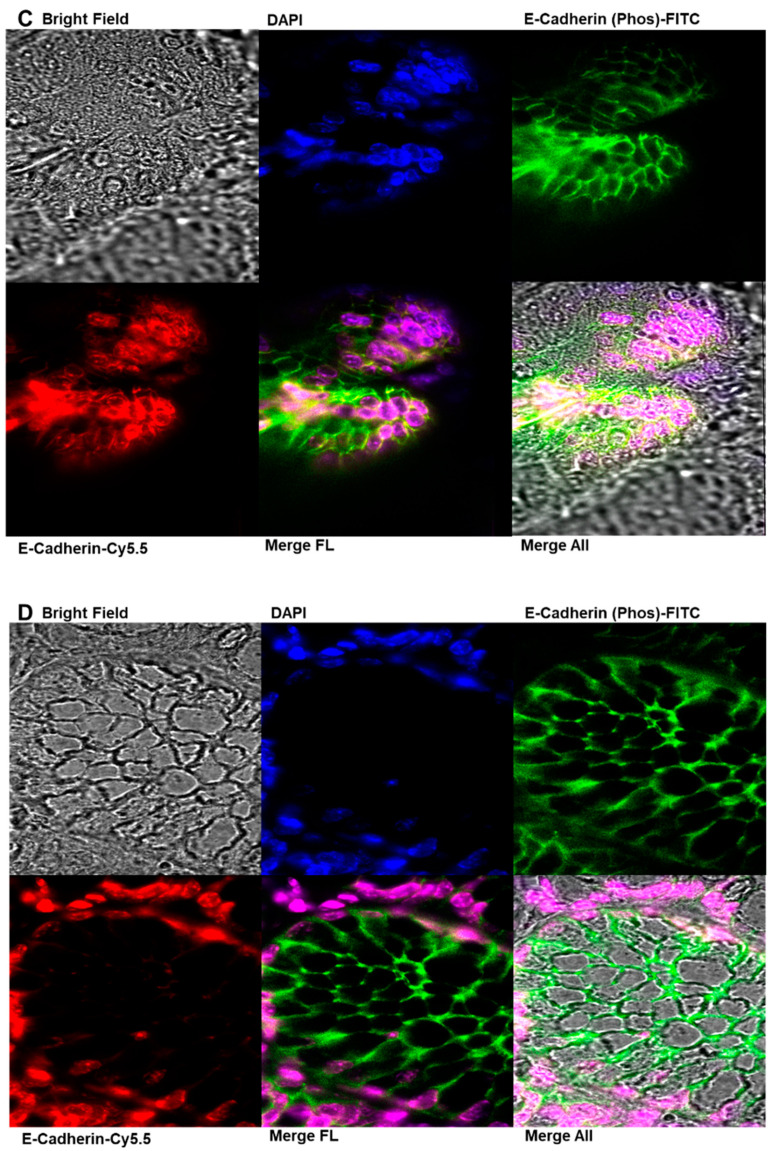

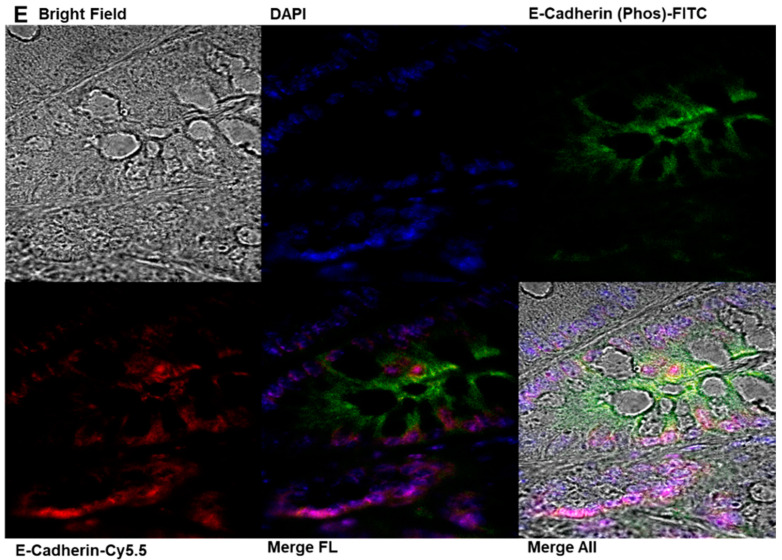
Immunofluorescence analysis of E-cadherin and P-cadherin localization in colonic epithelial tissues following AOM induction and FSE treatment. This figure presents representative immunofluorescence images illustrating expression and subcellular localization of E-cadherin and its phosphorylated form (S580 P-cadherin) across following experimental groups: (**A**) G1 (vehicle control), (**B**) G2 (AOM-only), (**C**) G3 (low-dose FSE + AOM), (**D**) G4 (high-dose FSE + AOM), and (**E**) G5 (FSE-only control). In AOM-treated group (G2), P-cadherin expression was markedly elevated, predominantly localized to cytoplasm and cell membrane and accompanied by epithelial thickening indicative of tumor-associated structural disruptions. Concurrently, E-cadherin exhibited abnormal cytoplasmic and nuclear localization, indicating compromised cell adhesion and potential loss of epithelial integrity. FSE treatment mitigated these alterations in dose-dependent manner, with G3 showing partial restoration of E-cadherin localization and G4 exhibiting enhanced nuclear E-cadherin expression, suggesting possible regulatory role in transcriptional activity and tumor suppression. Notably, localization of P-cadherin remained largely unaffected by FSE treatment. These results highlight disruptive effects of AOM on epithelial adhesion molecules, underscoring FSE’s potential protective role in maintaining epithelial structure and modulating carcinogenesis-related pathways. Confocal images were acquired using a 60× objective (NA = 1.40, oil immersion) on an Andor RD-DSD-600 confocal system, mounted on a Nikon TiE microscope.

Immunofluorescence analysis of colonic epithelial tissues revealed significant alterations in E-cadherin expression and subcellular localization and its phosphorylated form (phospho S838 + S840) following AOM induction and FSE treatment (Figure 7). In the AOM-treated group (G2), phosphorylated E-cadherin expression was markedly elevated, predominantly localizing to the cytoplasm and cell membrane. This upregulation, accompanied by epithelial thickening, suggests a disruptive effect of AOM on colonic epithelial architecture, consistent with pro-tumorigenic changes associated with colorectal carcinogenesis (Figure 7A,B,E). E-cadherin, a critical epithelial adhesion molecule typically localized to the plasma membrane to maintain cell–cell adhesion and epithelial polarity, exhibited an abnormal distribution in G2 (AOM-only group), with increased cytoplasmic and nuclear accumulation. FSE treatment demonstrated a dose-dependent modulatory effect on these disruptions. In the G3 group (low-dose FSE + AOM), E-cadherin expression showed partial restoration but remained primarily localized to the cytoplasm and nucleus, with limited membrane association. A more substantial restoration was observed in the G4 group (high-dose FSE + AOM), where nuclear localization of E-cadherin was significantly enhanced, accompanied by a reduction in cytoplasmic expression. Interestingly, the expression and localization of phosphorylated E-cadherin remained essentially unchanged across the FSE-treated groups, suggesting that FSE selectively influences the dynamics of unphosphorylated E-cadherin. These findings highlight AOM-induced disruptions in colonic epithelial adhesion mechanisms and demonstrate the potential of FSE to restore epithelial integrity and maintain cellular architecture by modulating E-cadherin localization.

### 2.8. Molecular Docking Analysis

**Figure 8:**
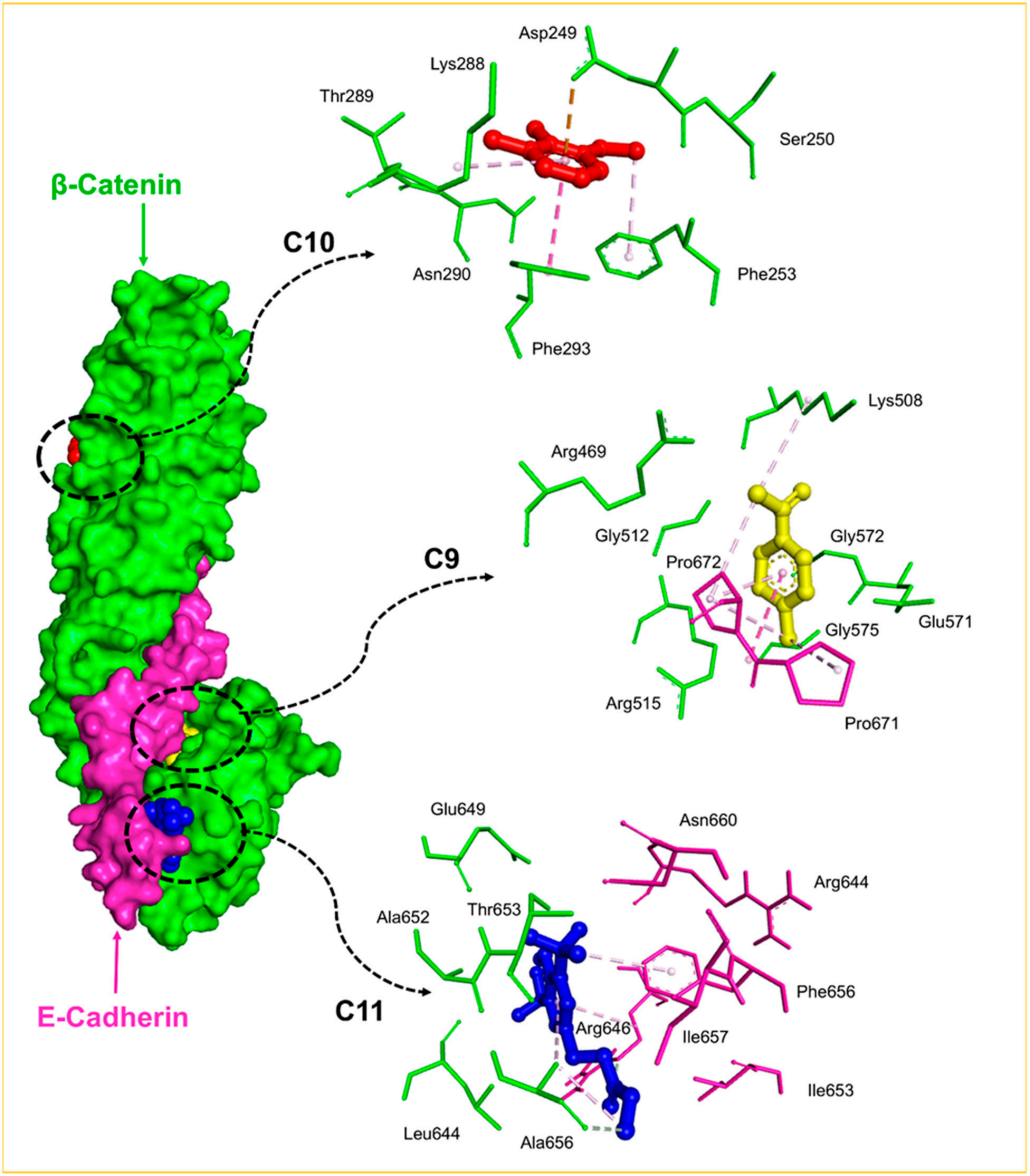
Molecular docking of fenugreek seed extract (FSE) compounds (C1–C11) with the E-cadherin–β-catenin protein complex. The protein complex is visualized using surface rendering, highlighting potential interaction sites. Key interacting residues of E-cadherin and β-catenin are represented as purple and green sticks, respectively. Representative docked compounds are displayed in a ball-and-stick format, with color coding to differentiate the three distinct binding regions: red for the first site, yellow for the second, and blue for the third. These binding sites reflect the specific interaction zones occupied by FSE bioactives, providing insights into their potential role in stabilizing the E-cadherin–β-catenin complex and influencing cell adhesion dynamics.

Following the immunofluorescence results, which revealed a significant upregulation and altered localization of E-cadherin in response to FSE treatment, a molecular docking analysis was conducted to explore how the bioactive compounds in FSE might interact with the E-cadherin–β-catenin complex. This approach aimed to elucidate the potential mechanisms by which FSE could help stabilize cell adhesion structures, restore epithelial integrity, and counteract tumor-promoting disruptions associated with CRC progression. Eleven bioactive compounds (C1–C11), identified through GC-MS analysis, were docked against the E-cadherin–β-catenin complex to predict their binding affinities and interaction sites. The analysis revealed three distinct binding regions within the complex (Figure 8), providing valuable insights into how these compounds may influence crucial protein–protein interactions that maintain epithelial cell cohesion. The results showed that compounds C1–C8 are predominantly bound to the β-catenin interface, with docking scores ranging from −5.50 kcal/mol to −5.95 kcal/mol. These interactions suggest that these compounds may stabilize the E-cadherin–β-catenin complex, thereby limiting the oncogenic signaling typically driven by β-catenin in cancer progression. Notably, C10 also targeted this region, with a slightly stronger binding affinity (−5.98 kcal/mol), indicating its potential role in disrupting β-catenin’s translocation and transcriptional activity—key drivers of tumor development. Compounds C9 and C11 exhibited high-affinity binding at two distinct interface regions within the E-cadherin–β-catenin complex, with scores of −5.59 kcal/mol and −6.72 kcal/mol, respectively. The notably strong interaction of C11 suggests that it may contribute significantly to stabilizing this protein complex, potentially reinforcing cell–cell adhesion and preventing the breakdown of epithelial integrity—a hallmark of epithelial-to-mesenchymal transition (EMT) and tumor progression. These findings provide compelling molecular evidence that bioactive compounds from FSE can strengthen the E-cadherin–β-catenin complex, thereby supporting epithelial barrier function and limiting β-catenin-mediated oncogenic signaling. This mechanistic insight complements the observed protective effects of FSE in the AOM-induced CRC model, highlighting its potential as a natural agent for cancer prevention and therapeutic support.

### 2.9. Molecular Dynamics Simulation

**Figure 9:**
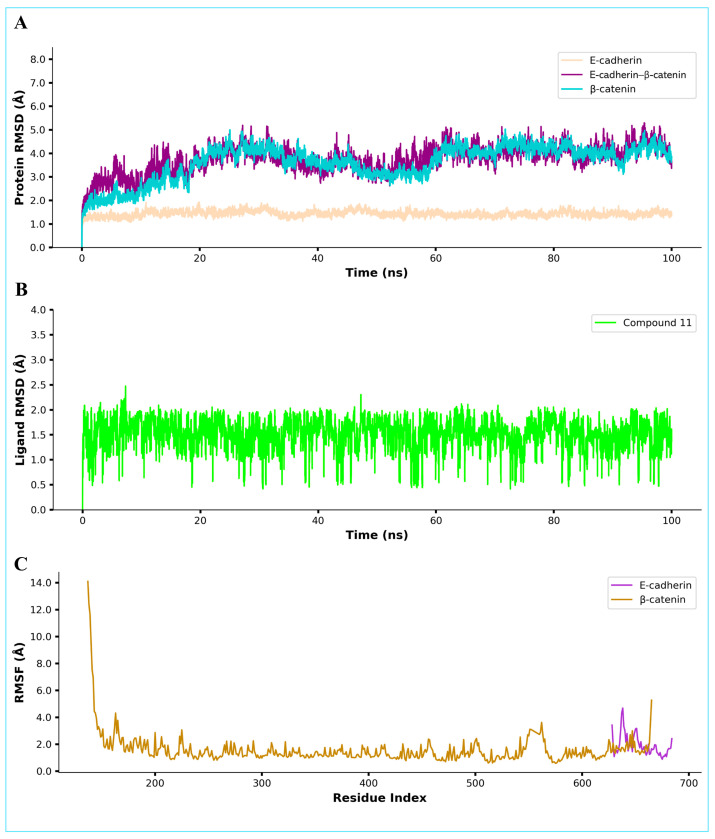
The results of the molecular dynamics simulation of fenugreek seed extract (FSE) compound (C11) in complex with the E-cadherin–β-catenin protein. (**Panel A**) shows the protein root-mean-square deviation (RMSD) plot, (**Panel B**) represents the ligand RMSD over 100 ns, and (**Panel C**) depicts the root-mean-square fluctuations (RMSF) of the amino acid residues during the simulation.

The stability of the docked complex under conditions closely reflecting a biological system was evaluated using a molecular dynamics (MD) simulation. This approach allowed for the observation of its conformational behavior and provided insights into its potential in vivo utility. Throughout the 100 ns simulation of compound 11 in an E-cadherin–β-catenin complex, E-cadherin maintained an RMSD of ~2 Å, indicating a relatively stable conformation. However, β-catenin and the entire ternary assembly exhibited slightly higher RMSD values, ranging from ~2.5 to 4 Å (Figure 9). These observations suggest that β-catenin contributes substantially to the overall conformational mobility of the complex.

Panel B of Figure 9 shows the ligand (Compound **11**) RMSD, which remains relatively stable between 1 and 2.5 Å, indicating a well-maintained binding mode throughout the trajectory. Furthermore, Panel C plots the residue-wise root-mean-square fluctuations (RMSF) of the β-catenin and E-cadherin proteins. β-Catenin exhibits a pronounced peak in the *N*-terminal region (up to ~14 Å), indicating significant mobility, followed by generally lower fluctuations for most of the structure, with a slight increase near the C-terminus. E-cadherin, on the other hand, exhibits overall moderate fluctuations, which align well with its lower overall RMSD and reinforce the picture of a comparatively stable protein structure.

**Figure 10:**
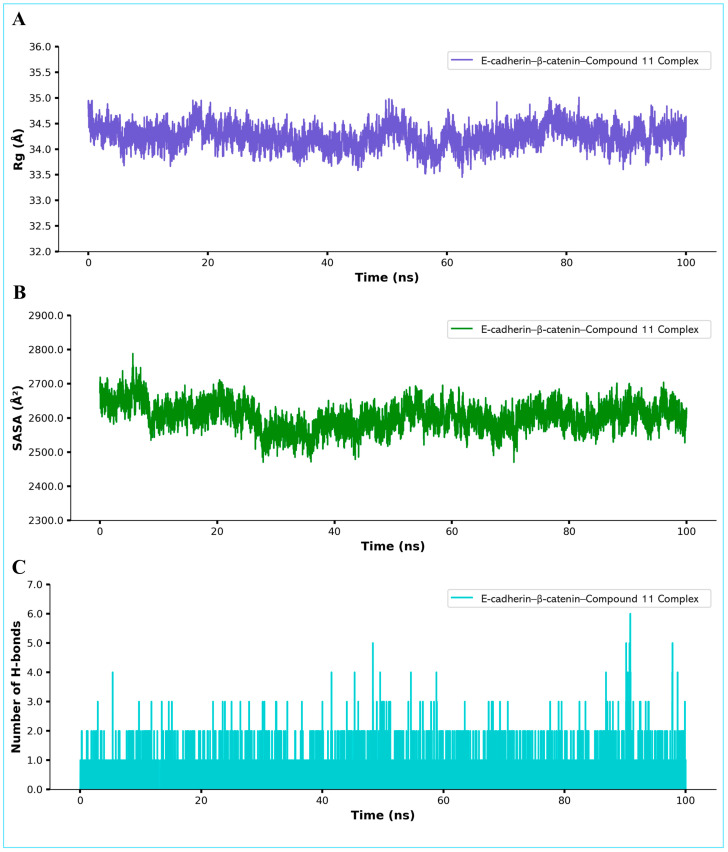
MD simulation analysis of Compound C11 in complex with E-Cadherin–β-Catenin protein, showing radius of gyration (**A**), solvent-accessible surface area (**B**), and number of hydrogen bonds observed (**C**) during 100 ns simulation.

The radius of gyration (Rg) analysis for the simulated complex, which remains fairly stable at around 34–35 Å throughout the 100 ns trajectory, further supports the stability of Compound 11 at the interface of E-cadherin and β-catenin (Figure 10; Panel A). This suggests that the overall fold and compactness of the ternary complex do not undergo large-scale rearrangements, consistent with a stable binding interaction. Analysis of the solvent-accessible surface area (SASA) is depicted in Panel B of Figure 10, which shows fluctuations between approximately 2300 and 2800 Å^2^. While some minor variations are observed, these SASA changes do not indicate major structural unfolding but rather typical breathing motions of the protein surface. The number of hydrogen bonds observed between Compound **11** and the E-cadherin–β-catenin interface is presented in Panel C of Figure 10, which shows consistent hydrogen bonding throughout the simulation, suggesting a recurring set of stabilizing interactions that may help anchor Compound 11 at the protein interface.

**Figure 11:**
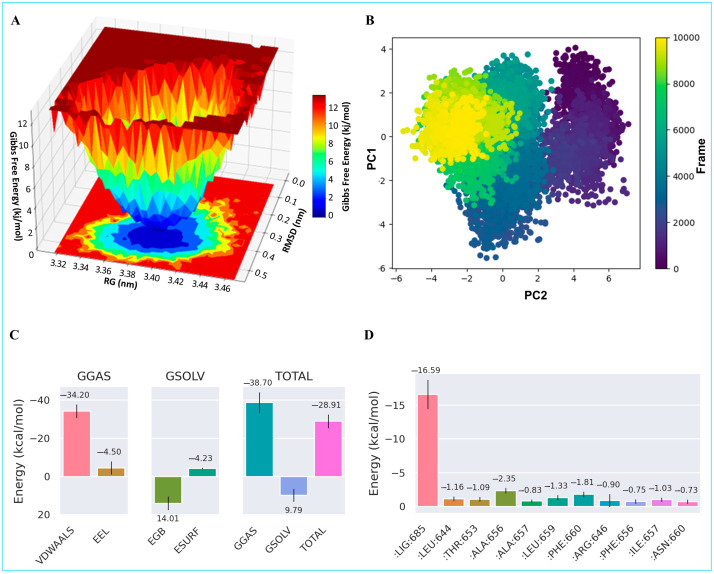
MD simulation analysis of Compound C11 in complex with E-Cadherin–β-Catenin protein, showing free energy landscape (FEL) in (**A**), principal component analysis (PCA) in (**B**), MM/GBSA binding energy in (**C**), and per-residue decomposition analysis in (**D**).

The free energy landscape (FEL) as a function of the radius of gyration (Rg) and RMSD is shown in Figure 11 (Panel A). The bottom of the energy well (shown in blue) indicates the most stable conformational basin around ~3.40 nm Rg and ~0.25–0.30 nm RMSD, suggesting a stable global minimum populated by the E-cadherin–β-catenin–Compound-11 assembly. Furthermore, a principal component analysis (PCA) plot of the MD frames projected onto the first two principal components (PC1 vs. PC2), with the frame index mapped onto a color scale, is demonstrated in Panel B (Figure 11). The distribution suggests that the simulation samples two primary conformational clusters, implying possible minor structural rearrangements or transitions between metastable states over the 100 ns timescale.

The binding affinity between Compound 11 and the protein complex was estimated using Molecular Mechanics/General Born Surface Area (MM/GBSA) calculations. The net binding free energy of −28.9 kcal/mol appears to be favorable, underscoring the potential of Compound 11 to stabilize the E-cadherin–β-catenin interface. Breaking down the total energy reveals that van der Waals interactions are the dominant driving force, with an energy of −34.2 kcal/mol (Figure 11, Panel C). However, electrostatic forces provide a smaller but still negative component of −4.50 kcal/mol. The solvation term offsets some of these attractive forces, but the net balance remains distinctly negative, underscoring the stability of the complex. Per-residue decomposition (Figure 11, Panel D) highlights that the Leu644, Thr653, Ala656, Leu659, Phe660, and Ile657 residues are the dominant contributors to the binding.

## 3. Discussions

This study provides compelling evidence supporting the chemopreventive potential of methanolic FSE in an AOM-induced CRC model. The findings demonstrate that FSE modulates essential cancer-related biomarkers, restores antioxidant defense mechanisms, and promotes apoptosis in transformed cells. Unlike previous studies primarily focused on fenugreek’s antioxidant and anti-inflammatory properties, this research comprehensively explores FSE’s multi-targeted actions, highlighting its potential as a natural therapeutic agent for CRC prevention. The high extraction yield (24%) achieved through methanolic cold maceration underscores the efficiency of this method in preserving bioactive compounds. Gas chromatography–mass spectrometry (GC-MS) analysis revealed 13 distinct bioactive constituents, including derivatives of benzene, cyclopentadiene, o-xylene, and benzenepropanoic acid, all of which have demonstrated antioxidant, anti-inflammatory, and cytotoxic activities [39,40,41,42]. Notably, some structurally similar compounds have been reported in previous phytochemical analyses of fenugreek, further supporting the relevance of our findings [36,43,44]. This diverse phytochemical profile likely contributes to the broad-spectrum chemopreventive effects observed in the FSE-treated groups. These findings align with earlier reports on the synergistic actions of plant-derived compounds in cancer prevention [45]. While GC-MS analysis provided a preliminary identification of bioactive compounds, further characterization using HPLC could offer deeper insights into the presence of steroidal saponins and other key constituents. Expanding phytochemical profiling with advanced analytical techniques will be valuable in confirming the primary active compounds responsible for FSE’s pharmacological effects. Acute toxicity testing confirmed FSE’s safety, reinforcing its potential for therapeutic application.

In the AOM-induced CRC model, FSE demonstrated strong protection against tumor progression, as shown by significant improvements in body weight maintenance and survival rates. Mice exposed to AOM experienced notable weight loss, a hallmark of cancer cachexia resulting from metabolic dysregulation, systemic inflammation, and heightened catabolic signaling [46,47]. FSE treatment mitigated these adverse effects in a dose-dependent manner, with high-dose administration (G4) fully restoring body weight and achieving 100% survival, compared to the 20% survival observed in the AOM-only group (G2) (Figure 2). These findings suggest that FSE counters cancer-associated metabolic imbalances and systemic inflammation, enhancing physiological resilience.

Serum biomarker analysis further supports FSE’s efficacy by demonstrating its ability to modulate key cancer-related enzymes associated with metabolic dysfunction and immune evasion. AOM exposure significantly elevated the levels of gamma-glutamyl transferase (GGT), 5′-nucleotidase (CD73), and lactate dehydrogenase (LDH), all of which are implicated in tumor metabolism and immune suppression. FSE treatment significantly reversed these changes in a dose-dependent manner. The marked reduction of CD73 levels suggests interference with purinergic signaling pathways, which are known to contribute to tumor immune evasion. Similarly, decreased LDH levels imply potential suppression of the Warburg effect, a metabolic hallmark of cancer cells that promotes glycolysis-driven tumor progression [48,49,50,51].

The antioxidative properties of FSE were also evident in the restoration of antioxidant enzyme activities. AOM exposure diminished the levels of superoxide dismutase (SOD), catalase (CAT), and glutathione peroxidase 1 (GPx1) while simultaneously increasing malondialdehyde (MDA), a marker of lipid peroxidation and oxidative damage [30,38,52,53]. FSE treatment significantly reversed these alterations in a dose-dependent manner, highlighting its potent antioxidative capacity. Restoring these enzymatic activities indicates that FSE protects cellular integrity by mitigating oxidative damage and preventing neoplastic transformation. Notably, FSE demonstrated comparable effectiveness to well-established antioxidants such as thymoquinone, 6-gingerol, and sulfur-containing compounds like diallyl trisulfide in restoring antioxidant defenses, positioning it as a promising and cost-effective natural alternative for CRC prevention [38,54,55]. Furthermore, recent studies on the methanolic extract of *Artemisia annua* have highlighted its chemopreventive potential, demonstrating a dose-dependent increase in apoptosis and preservation of normal tissue architecture [28]. These findings further underscore the therapeutic relevance of plant-derived bioactives in the prevention of CRC. FSE’s ability to reduce oxidative stress, modulate key biomarkers, and induce apoptosis reinforces its potential as a versatile chemopreventive agent in colorectal cancer management.

A notable observation was the biphasic effect of FSE on regulating oxidative stress. While FSE enhanced antioxidant defenses in normal cells, it simultaneously regulated ROS levels in transformed cells, leading to apoptosis induction. This dual-phase response reflects a well-documented mechanism among phytochemical-based cancer therapies. Flow cytometry analysis confirmed a dose-dependent increase in apoptotic cells within the FSE-treated groups, with apoptosis rates reaching 34.5% in the high-dose group (G4) (Figure 6). These findings suggest that FSE selectively promotes ROS-mediated apoptosis in carcinogen-exposed cells while safeguarding normal cellular architecture by modulating oxidative stress pathways [56,57,58].

To explore the molecular underpinnings of E-cadherin modulation, molecular docking analyses were conducted using 11 bioactive compounds (C1–C11) identified from the FSE. These compounds were docked against the E-cadherin–β-catenin complex, a critical regulator of cell–cell adhesion. Disruption of this interaction compromises epithelial cohesion, facilitating tumor progression. The docking results revealed three distinct binding regions within the complex, suggesting that multiple FSE compounds could stabilize E-Cadherin–β-catenin interactions. Compounds C1–C8 and C10 predominantly bound to β-catenin, with docking scores ranging from −5.5 kcal/mol to −5.98 kcal/mol, indicating moderate binding affinity.

In contrast, Compounds C9 and C11 targeted additional interface regions with higher binding affinities, particularly C11 (−6.72 kcal/mol), suggesting a more substantial potential for stabilizing the E-cadherin–β-catenin interaction. These findings suggest that FSE compounds could strengthen adherens junctions, potentially reinforcing epithelial integrity and limiting oncogenic signaling mediated by β-catenin [59]. The molecular docking findings complement the immunofluorescence data, supporting the hypothesis that FSE bioactives enhance E-cadherin expression and stabilize its interaction with β-catenin. By reinforcing cell–cell adhesion, these compounds may help maintain epithelial barrier function and inhibit processes associated with CRC progression, such as EMT. This mechanism highlights the therapeutic significance of FSE in restoring cellular architecture and inhibiting tumor development.

A molecular dynamics simulation was performed in this study to assess the stability and dynamic behavior of the docked complex under conditions that closely mimic a living system. By simulating the interaction between Compound C11 and the E-cadherin–β-catenin protein complex over time, we observed its conformational changes, flexibility, and key intermolecular interactions. The proteins maintain their general structural features, and the ligand binds in a manner that supports the interaction interface, chiefly via strong hydrophobic contacts. The balanced observations from RMSD, RMSF, Rg, SASA, FEL, PCA, and MM/GBSA underscore that Compound 11 may effectively stabilize the E-cadherin–β-catenin complex, supporting its potential role in preserving cell–cell adhesion.

These computational findings provided the foundation for subsequent immunofluorescence analysis, which further examined the role of FSE in regulating E-cadherin expression and its association with apoptotic pathways. Immunofluorescence analysis revealed that AOM-treated tissues exhibited altered E-cadherin localization, with increased cytoplasmic and nuclear accumulation, indicative of epithelial-to-mesenchymal transition (EMT), a process associated with cancer progression. The loss of membrane-bound E-cadherin weakens cell–cell adhesion and compromises epithelial integrity [60]. FSE treatment dose-dependently restored E-cadherin localization at the membrane, particularly in the high-dose group (G4), reinforcing epithelial cohesion and tumor-suppressive functions. The re-establishment of E-cadherin at the membrane aligns with its well-recognized role in maintaining epithelial integrity and preventing oncogenic dedifferentiation [61].

The pro-apoptotic effects observed in the FSE-treated groups may be attributed to multiple interconnected signaling cascades [62,63]. Loss of E-cadherin has been associated with increased nuclear translocation of β-catenin, which activates Wnt signaling and promotes tumorigenesis. Restoration of E-cadherin through FSE treatment may sequester β-catenin at the membrane, limiting its nuclear activity and attenuating Wnt-driven transcriptional regulation of oncogenic targets [64,65]. Additionally, the downregulation of E-cadherin is known to modulate p53 activity, leading to increased expression of apoptotic mediators such as Bax and PUMA. Stabilizing E-cadherin following FSE treatment may contribute to p53-dependent apoptosis, reinforcing tumor suppression mechanisms [66,67]. Furthermore, E-cadherin loss has been implicated in YAP/TAZ activation, a pathway associated with enhanced proliferation and survival. FSE-mediated restoration of E-cadherin may restrict YAP nuclear localization, shifting the cellular response towards pro-apoptotic signaling, possibly via TP73-mediated pathways [68,69]. Reinforcing E-cadherin-mediated adherens junctions may also contribute to the activation of caspase-9, further promoting apoptosis in neoplastic cells [66]. Another possible pathway involves NF-κB signaling, where E-cadherin loss has been linked to the activation of NF-κB, a key regulator of inflammatory and survival pathways. Restoring E-cadherin expression, FSE may inhibit NF-κB activation, attenuating inflammation-induced tumorigenesis and shifting the cellular balance toward apoptosis [70,71].

Interestingly, phosphorylated E-cadherin (phospho S838 + S840) levels remained unchanged following FSE treatment, suggesting that FSE selectively influences the dynamics of unphosphorylated E-cadherin. This observation suggests that FSE enhances E-cadherin expression while preserving its functional role, thereby ensuring stability within cell–cell adhesion complexes. The modulation of key oncogenic pathways by FSE aligns with previous findings on other phytochemicals known for their chemopreventive effects. Resveratrol has been shown to inhibit NF-κB activation by suppressing protein kinase C-β2 (PKC-β2) phosphorylation, reducing downstream target gene expression, including iNOS, COX-2, and aldose reductase in AOM-stimulated mouse colon tissues [72]. Similarly, curcumin exerts its tumor-suppressive effects by downregulating the Wnt/β-catenin signaling pathway, a key pathway frequently dysregulated in CRC progression [73]. The ability of FSE to modulate multiple signaling pathways, such as those of these well-established phytochemicals, underscores its potential as a viable candidate for CRC chemoprevention. These findings suggest that FSE may function by restoring E-cadherin expression and stabilizing its interaction with key adhesion and signaling molecules, thereby contributing to the structural and functional integrity of epithelial tissue. These findings suggest that FSE functions by restoring E-cadherin expression, stabilizing its interaction with key adhesion and signaling molecules, and preserving the structural and functional integrity of epithelial tissue. Understanding its mechanistic similarities with established chemopreventive agents provides valuable insights into FSE’s translational potential in CRC prevention strategies.

Building on these mechanistic insights, FSE emerges as a promising natural chemopreventive agent with a multifaceted mechanism of action, including the modulation of cancer biomarkers, restoration of antioxidant defenses, regulation of oxidative stress, induction of apoptosis, and stabilization of cell adhesion dynamics. Unlike conventional therapies that target a single oncogenic pathway, FSE exerts broad-spectrum effects, positioning it as a viable candidate for CRC prevention strategies. Beyond its chemopreventive properties, emerging evidence suggests that FSE may also function as a chemosensitizing agent, enhancing the efficacy of conventional chemotherapeutics by modulating apoptotic and survival pathways. Exploring its role in improving therapeutic responses while reducing the toxicity of standard treatments, particularly through metronomic dosing strategies, could be a valuable avenue for future research.

Additionally, considering the critical role of the gut microbiome in CRC pathogenesis, it is an important aspect to explore whether FSE exerts its protective effects, at least in part, by influencing microbial composition and function. The gut microbiota is fundamental in regulating inflammation, metabolic homeostasis, and immune responses, all intricately linked to colorectal carcinogenesis [74,75]. Investigating the impact of FSE on gut microbial dynamics may provide further insights into its mechanisms of action and expand its translational relevance in CRC prevention.

As the first study of its kind, this research provides a comprehensive investigation into the chemopreventive potential of FSE, integrating both preclinical validation and computational insights. We extensively explored the molecular mechanisms underlying FSE’s effects through molecular docking and immunofluorescence analyses, offering mechanistic evidence supporting its role in colorectal cancer prevention. Future research should further elucidate the pathways by which FSE bioactives regulate E-cadherin-mediated apoptotic signaling and assess their ability to optimize the tumor microenvironment and modulate the immune response. With its demonstrated efficacy in restoring epithelial integrity, mitigating oxidative stress, and inducing apoptosis, FSE emerges as a promising and accessible chemopreventive agent with significant translational potential for the treatment of CRC. These findings lay the foundation for future studies to explore its therapeutic applications and mechanistic intricacies in greater depth.

## 4. Methods

### 4.1. Materials

AOM was obtained from Sigma-Aldrich (St. Louis, MO, USA). Reagents and assay kits were primarily sourced from Abcam (Cambridge, MA, USA), including the Annexin V-FITC Apoptosis Staining/Detection Kit (ab14085), 2′,7′-dichlorofluorescin diacetate (DCFDA/H2DCFDA) Cellular ROS Assay Kit (ab113851), Lactate Dehydrogenase (LDH) Activity Assay Kit (ab102526), Gamma-Glutamyl Transferase (γ-GT) Assay Kit (ab241029), Superoxide Dismutase (SOD) Assay Kit (ab65354), Catalase (CAT) Assay Kit (ab83464), Lipid Peroxidation (MDA) Assay Kit (ab233471), and Glutathione Peroxidase 1 (GPx1) Assay Kit (ab10250). Primary antibodies used included anti-E-cadherin [4A2] and E-cadherin (phospho S838 + S840) antibody [EP913(2)Y]. For secondary detection, a Biotin-conjugated Goat Anti-Mouse IgG H&L antibody (ab6720; Abcam) was used, followed by visualization using recombinant streptavidin-Cy5 (ab279319; Abcam) and streptavidin-FITC (ab2777701). Nuclear staining was performed using a Mounting Medium with DAPI (ab104139; Abcam). The 5′-Nucleotidase/CD73 Activity Assay Kit (Colorimetric) Catalog # NBP3-24452 was purchased from Tocris Bioscience (Bio-Techne) in Bristol, UK. Fenugreek seeds were sourced from a local organic market in Buraydah, Saudi Arabia. All additional chemicals and laboratory consumables were acquired from local suppliers to ensure consistent quality and experimental reproducibility.

### 4.2. Methanolic Extraction of Fenugreek Seeds (FSE) Using Cold Maceration Technique

The cold maceration technique was employed to preserve heat-sensitive bioactive compounds. Methanol was used as the solvent for its efficiency in extracting a wide range of phytochemicals. The extraction protocol was adapted from previously established methods with minor modifications [28]. Three hundred grams of dried fenugreek seeds were ground into coarse powder and defatted using 900 mL of cyclohexane. The mixture was stirred for four hours and centrifuged at 5000 rpm for 10 min. The impurity supernatant was discarded, and the defatted powder was air-dried at 40 °C overnight. The dried material was extracted with 900 mL of pure methanol for 72 h under ambient conditions. The resulting mixture was filtered through Whatman No. 1 filter paper, and the filtrate was concentrated using a rotary evaporator under a nitrogen atmosphere, yielding a dry, powdered extract.

### 4.3. GC-MS (Gas Chromatography–Mass Spectrometry) Analysis

The bioactive compounds in the FSE were analyzed using gas chromatography–mass spectrometry (GC-MS) with an Agilent 7890A GC system coupled to a 5975C triple-axis detector. Compound separation was achieved using an HP-5MS capillary column (30 m × 0.25 mm × 0.25 µm film thickness) with helium as the carrier gas at a flow rate of 1 mL/min.

The injector temperature was set to 230 °C, with a splitless injection mode (split ratio: 20:1). The column temperature was programmed to start at 40 °C, followed by a linear ramp to 150 °C at 10 °C/min, and finally to 300 °C, with a 1 min hold at each stage. The mass spectrometer operated at an ion source temperature of 150 °C, with electron ionization at 70 eV. The resulting spectra were compared against the National Institute of Standards and Technology (NIST) 2008 spectral library to facilitate compound identification. This optimized approach enabled accurate and detailed chemical profiling of FSE, providing valuable insights into its potential bioactive properties.

### 4.4. Molecular Docking Studies

The 3D coordinates of eleven FSE compounds were retrieved from the PubChem database. Each compound was processed using MGLTools 1.5.7 to merge nonpolar hydrogens and ascertain rotatable bonds and torsion trees. Gasteiger charges were assigned to every atom before saving the structures in pdbqt format [76]. The three-dimensional structure of the E-cadherin–β-catenin complex was obtained from the Protein Data Bank (http://www.rcsb.org/pdb/home/home.do, accessed on 27 January 2025) with pdb code of 1I7X to serve as a receptor [59]. The protein was prepared in Biovia Discovery Studio Visualizer 2021 by checking for missing residues or atoms and removing co-crystallized water molecules. A grid box with 1 Å spacing was set up to cover the entire E-cadherin–β-catenin protein complex, encompassing 84, 60, and 106 points along the *x*, *y*, and *z* axes, respectively, with a grid center at 38.708, 0.035, and 15.552. Blind docking was performed using AutoDock Vina v1.2 [77], generating twenty conformations per compound. The docking simulations were conducted with default settings, keeping the ligands flexible while maintaining the rigidity of the protein structures. The optimal ligand poses were chosen based on the binding energy (ΔG_binding_, kcal/mol), and each ligand–protein complex was subsequently analyzed for molecular interactions using Biovia Discovery Studio Visualizer 2021.

### 4.5. Molecular Dynamics Simulation Studies

The complex formed between Compound C11 and the E-cadherin–β-catenin protein was subjected to comprehensive all-atom molecular dynamics (MD) simulations using GROMACS version 2022.4 [78]. Briefly, the protein–ligand complex was submerged in a cubic simulation box with a 10 Å edge distance filled with TIP3P water molecules. The system charge was balanced by adding an appropriate number of Na^+^ and Cl^−^ ions using 0.15 M NaCl concentration. The system was prepared using the Stage3 tool [79], employing the AMBER99SB-ILDN and GAFF2 force fields for protein and ligand, respectively. Ligand charge calculation was performed using the AM1-BCC method. To eliminate steric clashes, the system was energy-minimized using a step size of 0.01 and 50,000 steps of steepest descent to achieve optimal convergence. This was followed by 1 ns of NVT and NPT equilibration phases at 310.15 K and 1 bar, utilizing the modified Berendsen thermostat and Parrinello–Rahman barostat, respectively. The system, once equilibrated, was subjected to a 100 ns production run under NPT conditions at 310.15 K and 1 bar. Long-range electrostatics and hydrogen bond constraints were handled using the PME [80] and LINCS [81] algorithms, respectively, with a time step of 2 fs. Finally, trajectory analyses were conducted using standard GROMACS v2022.4 tools, including RMSD, RMSF, radius of gyration, SASA, and hydrogen bonds, to evaluate the stability and interactions of the system [82].

### 4.6. MM/GBSA Binding Energy Computations

The calculation of binding free energies for Compound **11** was performed using the Molecular Mechanics/Generalized Born Surface Area (MMGBSA) method, as outlined by Miller et al. [83], and the gmx_MMPBSA tool version 1.6.2, developed by Valdés-Tresanco et al. [84]. The method involved extracting 400 frames from the final 40 nanoseconds of the MD simulation, taken at an interval of 10 picoseconds, to ensure a metastable sample for analysis. The binding free energy (ΔG_total_) of a ligand to its receptor was determined by calculating the difference between the free energy of the receptor–ligand complex and the sum of the free energies of the receptor and ligand. This approach takes into account various energetic contributions to the total free energy, including the gas phase energy (GGAS) driven by van der Waals (VDWAALS) and electrostatic interactions (EEL), as well as the solvation energy (GSOLV), which is further broken down into polar (EGB or EPB) and nonpolar (ESURF or ENPOLAR) components [83]. Additionally, the contribution of each residue to the binding free energy was also calculated.

### 4.7. In Vivo Studies

#### 4.7.1. Mice

Female Swiss albino mice (8–10 weeks old) were obtained from the Animal Research Facility at King Saud University, Riyadh, Saudi Arabia. The study protocol (24-97-02) was approved on 2 July 2024, by the Animal Ethics Committee of Qassim University, and all experimental procedures were conducted in strict adherence to institutional and international guidelines for the care and use of animals.

The mice were housed under standard laboratory conditions in the CAMS animal facility, with unrestricted access to food and water (ad libitum). Training staff performed twice-daily health checks throughout the study to ensure animal welfare and minimize distress. Humane euthanasia was carried out using CO_2_ inhalation, and no pre-euthanasia deaths were recorded during the experiment.

#### 4.7.2. Experimental Design

**Figure 12:**
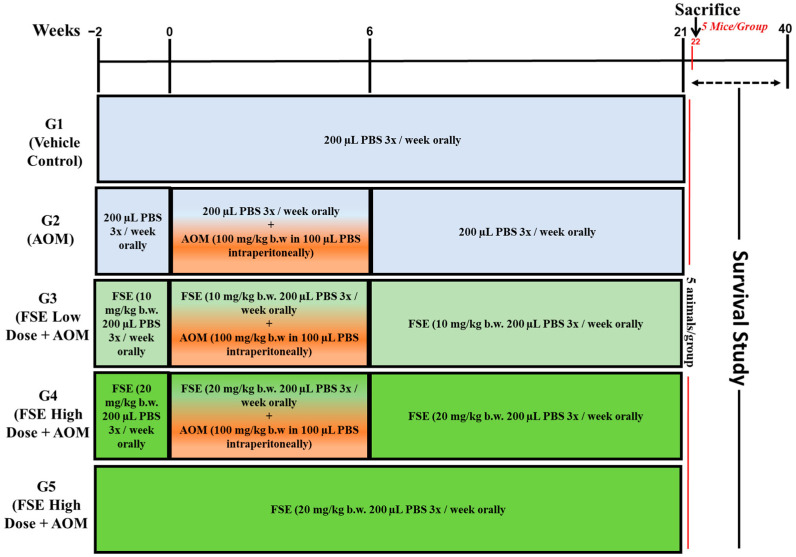
Experimental design overview. This schematic representation illustrates study design and treatment regimens followed for all experimental groups. G1 (Vehicle Control) received phosphate-buffered saline (PBS) orally thrice weekly from week −2 to week 21. G2 (AOM-treated group) was given azoxymethane (AOM) intraperitoneally at dose of 10 mg/kg body weight in 200 µL PBS three times per week from week 0 to week 6. G3 (FSE Low Dose + AOM) received 10 mg/kg FSE orally in PBS from week −2 to week 21, combined with AOM administration, as in G2. G4 (FSE High Dose + AOM) was treated orally with 20 mg/kg FSE in PBS from week −2 to week 21, alongside AOM exposure, as in G2. G5 (FSE High Dose Control) received 20 mg/kg FSE orally in PBS from week −2 to week 21 without AOM exposure. PBS and FSE were administered orally across all groups, whereas AOM was administered intraperitoneally according to designated schedule. At week 22, five mice from each group were sacrificed for biochemical and histopathological analyses, while remaining ten mice per group continued in study for survival analysis until week 40.

Seventy-five Swiss albino mice were randomly divided into five experimental groups (*n* = 15 per group), as detailed in the schematic illustration provided in Figure 12. The control group (G1) received phosphate-buffered saline (PBS) orally from week −2 through week 21. The AOM-treated group (G2) received intraperitoneal injections of AOM at a dose of 10 mg/kg body weight three times a week for six weeks to induce colorectal carcinogenesis. G3 received a low dose of FSE (10 mg/kg) orally from week −2 to week 21, concurrently with AOM treatment following the same schedule as G2. G4 was given a high dose of FSE (20 mg/kg) under the same regimen alongside AOM exposure. To evaluate the safety profile of FSE under normal physiological conditions, G5 was treated with 20 mg/kg of FSE without AOM exposure. At week 22, five mice from each group were sacrificed for biochemical and immunohistological analyses. The remaining mice (*n* = 10 per group) were monitored for survival analysis. Animal welfare was ensured in accordance with institutional guidelines, with any mice exhibiting signs of distress or reduced responsiveness being humanely euthanized within 24 h. Survival data were collected until week 40.

### 4.8. Study of Average Body Weight (ABW) and Survival Rate

The average body weight (ABW) of each group was recorded at week 0 and monitored every two weeks for a total of 22 weeks. Survival rates were tracked for 18 weeks after the initial AOM treatment, concluding at week 40.

### 4.9. Effect of FSE on Serum Cancer Markers

Serum levels of cancer markers, including lactate dehydrogenase (LDH), gamma-glutamyltransferase (γ-GT), and 5′-nucleotidase (5′-NT), were measured using assay kits from ABCAM. The assays were conducted according to the manufacturer’s instructions.

### 4.10. Effect of FSE on Colonic Antioxidant Enzyme Levels

The activities of key antioxidant enzymes, superoxide dismutase (SOD), catalase (CAT), malondialdehyde (MDA), and glutathione peroxidase 1 (GPx1), were evaluated using commercially available assay kits, following the manufacturer’s protocols. For the analysis, approximately 25 mg of colonic tissue was carefully weighed and homogenized in 100 µL of the respective buffer supplied with each assay kit. The homogenates were centrifuged at 10,000× *g* for 15 min at 4 °C, and the resulting supernatants were collected for biochemical analysis. The enzyme activities were then measured according to the kit instructions, ensuring consistency across all samples. The assays provided quantitative assessments of the enzymatic activities, enabling an evaluation of FSE’s protective effects against oxidative stress in AOM-exposed colonic tissues.

### 4.11. Annexin V-FITC/PI Apoptosis Assay and Reactive Oxygen Species (ROS) Detection

Colon tissues were dissociated into a single-cell suspension using the gentleMACS Dissociator (Miltenyi Biotec, Bergisch Gladbach, Germany) and passed through a 100 µm mesh to eliminate debris and aggregates. The resulting suspension was centrifuged at 300× *g* for 10 min at 4 °C, and the cell pellet was resuspended in an appropriate buffer for further analysis.

For ROS detection, cells were incubated with 20 µM 2′,7′-dichlorofluorescein diacetate (DCFDA) at 37 °C for 40 min in the dark. DCFDA is oxidized into a fluorescent compound upon interaction with intracellular ROS, allowing quantitative assessment of ROS levels. Fluorescence intensity was measured using the MACSQuant Analyzer 10 (Miltenyi Biotec, Bergisch Gladbach, Germany), and mean fluorescence intensity (MFI) values were calculated using FlowJo software version 10.9.0. Histogram plots were generated to visualize the distribution of ROS among the experimental groups.

For apoptosis detection, cells were stained with Annexin V-FITC and propidium iodide (PI) in a binding buffer, following the manufacturer’s protocol. This enabled differentiation between viable, early apoptotic, late apoptotic, and necrotic cells. After 20–25 min of incubation at room temperature in the dark, samples were analyzed using the MACSQuant Analyzer 10. Data were processed using FlowJo software (version 10.8.1) to generate cell distribution plots and quantify apoptotic populations across experimental groups.

### 4.12. Immunofluorescence Analysis of E-Cadherin and Phosphorylated E-Cadherin Localization

The expression, subcellular localization, and phosphorylated form (phospho S838 + S840) of E-cadherin were assessed using immunofluorescence in colonic epithelial tissues. Following euthanasia, colon samples were collected, rinsed with Tris-buffered saline (TBS), and fixed in 4% paraformaldehyde for 24 h at 4 °C. The tissues were dehydrated through a graded ethanol series, cleared with xylene, and embedded in paraffin. Sections of 10 µm thickness were prepared using a rotary microtome and underwent antigen retrieval in 10 mM citrate buffer (pH 6.0) at 95 °C for 20 min. Non-specific binding was blocked with 5% bovine serum albumin (BSA) in TBS containing 0.1% Triton X-100 for 1 h at room temperature. Sections were first incubated overnight at 4 °C with a monoclonal anti-E-cadherin [4A2] primary antibody (1:200 dilution; Abcam, Cambridge, UK). After washing with TBS, slides were treated with an anti-mouse Biotin-conjugated secondary antibody (Goat Anti-Mouse IgG H&L; ab6720, Abcam, Cambridge, UK) and subsequently incubated with recombinant streptavidin-Cy5 to visualize E-cadherin. For sequential staining, sections were incubated with an E-cadherin (phospho S838 + S840) antibody [EP913(2)Y] (1:200 dilution) for 2 h at room temperature. After washing, sections were re-treated with the same anti-mouse Biotin-conjugated secondary antibody, followed by incubation with recombinant streptavidin-FITC for 20 min. Nuclear staining was performed using a Mounting Medium with DAPI. Fluorescence imaging was performed using the Andor RD-DSD-600 confocal system coupled with a Nikon TiE microscope (Tokyo, Japan). Imaging parameters were standardized across all groups, and localization analysis of membrane, cytoplasmic, and nuclear compartments was conducted using ImageJ 1.53k software.

### 4.13. Statistical Analysis

All data were expressed as the mean with corresponding 95% confidence intervals (CI). Comparisons between experimental groups were conducted using one-way or two-way analysis of variance (ANOVA), followed by Tukey’s post hoc test for multiple comparisons. Statistical analyses were performed using GraphPad Prism 9. A *p*-value of <0.05 was considered statistically significant.

## 5. Conclusions

This study highlights the promising chemopreventive potential of methanolic FSE in an AOM-induced CRC model. FSE effectively modulated key cancer biomarkers, restored antioxidant defenses, and induced apoptosis in transformed cells. Molecular docking revealed strong interactions between FSE-derived bioactives and the E-cadherin–β-catenin complex, supporting its role in stabilizing cell adhesion and limiting tumor progression. Additionally, FSE’s ability to reinforce epithelial integrity and modulate oxidative stress underscores its therapeutic potential. Given its low toxicity and multifaceted mechanisms, FSE emerges as a viable, natural adjunct for CRC prevention, warranting further clinical investigation and the exploration of synergistic effects with conventional therapies.

## Figures and Tables

**Table 1:** Bioactive compounds identified in methanolic fenugreek seed extract (FSE) via cold maceration method.

Compound Number	RT (min)	Scan Number (#)	Area (Ab·s)	Baseline Height (Ab)	Absolute Height (Ab)	Peak Width 50% (min)	Hit Number	Hit Name	Quality	Mol Weight (amu)
**1**	5.05	343	182,804	29,865	34,215	0.16	1	Benzene, 1,3-dimethyl-	94	106.078
**2**	5.427	409	28,045	7262	12,506	0.086	1	1,3-Cyclopentadiene, 5-(1-methylethylidene)-	94	106.078
**3**	5.502	422	103,925	22,117	25,147	0.217	1	o-Xylene	95	106.078
**4**	5.948	500	8311	3799	6860	0.063	1	Benzene, (1-methylethyl)-	43	120.094
**5**	6.457	589	42,947	8712	12,028	0.126	1	Benzene, propyl-	80	120.094
**6**	6.812	651	20,495	8476	20,988	0.057	1	Benzene, 1-ethyl-2-methyl-	93	120.094
**7**	7.098	701	549,840	75,785	83,135	0.338	1	Benzene, 1,2,3-trimethyl-	95	120.094
**8**	7.487	769	20,667	9854	17,249	0.057	1	Benzene, 1-ethyl-3-methyl-	90	120.094
**9**	8.036	865	25,453	6644	15,658	0.097	1	1,3,8-p-Menthatriene	52	134.11
**10**	9.049	1042	51,010	14,409	22,019	0.109	1	Benzene, 1-ethyl-2,3-dimethyl-	94	134.11
**11**	13.158	1760	41,098	12,250	13,143	0.154	1	Tridecane	59	184.219
**12**	16.127	2279	66,807	8267	9759	0.223	1	Dotriacontane	72	450.516
**13**	20.115	2976	206,276	43,730	46,268	0.24	1	Benzenepropanoic acid, 3,5-bis(1,1-dimethylethyl)-4-hydroxy-, methyl ester	93	292.204

## Data Availability

The original contributions presented in this study are included in the article. Further inquiries can be directed to the corresponding author.
